# TNF signaling and macrophages govern fin regeneration in zebrafish larvae

**DOI:** 10.1038/cddis.2017.374

**Published:** 2017-08-10

**Authors:** Mai Nguyen-Chi, Béryl Laplace-Builhé, Jana Travnickova, Patricia Luz-Crawford, Gautier Tejedor, Georges Lutfalla, Karima Kissa, Christian Jorgensen, Farida Djouad

**Affiliations:** 1 IRMB, INSERM, Univ Montpellier, Montpellier, France; 2 DIMNP, CNRS, Univ Montpellier, Montpellier, France; 3 Laboratorio de Immunologia Celular y Molecular, Facultad de Medicina, Universidad de Los Andes, Santiago, Chile; 4 Clinical Unit for Osteoarticular Diseases and Department for Biotherapy, CHU Lapeyronie, Montpellier, France

## Abstract

Macrophages are essential for appendage regeneration after amputation in regenerative species. The molecular mechanisms through which macrophages orchestrate blastema formation and regeneration are still unclear. Here, we use the genetically tractable and transparent zebrafish larvae to study the functions of polarized macrophage subsets during caudal fin regeneration. After caudal fin amputation, we show an early and transient accumulation of pro-inflammatory macrophages concomitant with the accumulation of non-inflammatory macrophages which, in contrast to pro-inflammatory macrophages, remain associated to the fin until the end of the regeneration. Chemical and genetic depletion of macrophages suggested that early recruited macrophages that express TNF*α* are critical for blastema formation. Combining parabiosis and morpholino knockdown strategies, we show that TNF*α*/TNFR1 signaling pathway is required for the fin regeneration. Our study reveals that TNFR1 has a necessary and direct role in blastema cell activation suggesting that macrophage subset balance provides the accurate TNF*α* signal to prime regeneration in zebrafish.

In contrast to mammals, lower vertebrates including zebrafish (*Danio rerio*) have the fascinating potential to regenerate lost or damaged tissues such as caudal fin.^1, 2, 3, 4^ This phenomenon, called epimorphic regeneration, occurs through the formation of a structure called blastema made of an accumulation of highly proliferative stromal cells that proliferate and differentiate to form the exact copy of the lost structure.^5^ Although the mechanisms underlying blastema formation and epimorphic regeneration are widely investigated they are still poorly understood. Wound fate relies on molecular and cellular processes that share common features among the animal species. Immediately after any injury, immune cells infiltrate the wound.^6, 7, 8^ Although leukocytes are indispensable to fight microbial invasion, they are also thought to have pivotal functions in the injury outcomes, that is, fibrosis *versus* regeneration.

Recently, much attention has been devoted to the role of macrophages in epimorphic regeneration. Initially described for their phagocytosis capacity, macrophages have a central role in inflammation, but also participate actively in its resolution. Macrophages exert both beneficial functions in development, homeostasis and tissue repair, and detrimental roles in some human diseases.^9^ In adult axolotls, macrophages have been shown to be required for limb regeneration after amputation.^10, 11^ Depletion of macrophages at different time points of the regeneration process using liposome encapsulated clodronate revealed that early macrophages are critical for this process.^10^

In zebrafish larvae, studies focusing on the role of macrophages in caudal fin regeneration using morpholino strategy and mutants led to controversial results. Indeed, *Pu.1* morpholino (MO) inhibiting the development of all myeloid lineage does not affect fin regeneration, whereas interferon regulatory factor 8 (*irf8*) MO, which blocks the differentiation of macrophage lineage was reported to either delay or produce no effect on fin regeneration.^7, 12, 13^ In zebrafish *cloche* mutants, which lack most hematopoietic tissues and cells, including the myeloid ones, caudal fin regeneration is initiated but increased apoptosis and absence of regenerative cell proliferation are observed leading to regeneration defects.^14^ These discrepancies might be due to the difficulty to specifically and efficiently suppress macrophage lineage without affecting other cell types and underline the need of an accurate method to study their role during larval regeneration. In adult zebrafish, genetic ablation of macrophages during the whole time course of regeneration affects the rate of caudal fin regeneration by impairing blastema cell proliferation, whereas ablation during the tissue outgrowth phase only changes the tissue patterning.^15^ This stage-dependent effect of macrophages on epimorphic regeneration strongly suggests the involvement of distinct functional populations of macrophages orchestrating the different phases of limb/fin regeneration. However, this has never been clearly demonstrated.

Recently, we have shown the existence of different macrophage subtypes in the zebrafish. Injury or *E. coli* infection induced the polarization of macrophages toward M1- and M2-like macrophage phenotypes expressing pro-inflammatory cytokines such as *tumor necrosis factor alpha* (*tnfa*), *interleukin-6* (*il6*) and *il1b* and anti-inflammatory cytokines such as *transforming growth factor beta-1* (*tgfb1*).^16^ These macrophage subsets are similar to the classically activated M1 and alternatively activated M2 macrophages described in mammals.^17, 18, 19, 20^ In the present study, we used the zebrafish larvae as a tractable system to (1) study the recruitment of functionally distinct subpopulations of macrophages to the wound after caudal fin amputation, (2) investigate their role during the individual phases of epimorphic regeneration and (3) identify the macrophage-derived molecule that promotes a regeneration-permissive environment.

## Results

### Macrophage recruitment and activation during caudal fin regeneration

**Figure 1:**
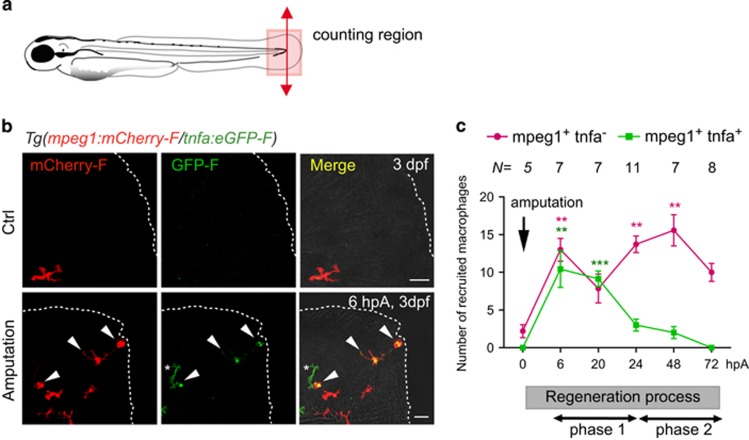
*Tnfa*^+^ and *tnfa*^−^ macrophages are differentially recruited during caudal fin regeneration in zebrafish larvae. (**a**) Diagram showing the amputation plan (red arrow) and the counting region (pink box) in the 3  dpf larvae. (**b**) Caudal fin of *Tg(mpeg1:mCherry-F/ tnfa:eGFP-F)* were amputated at 3 dpf as shown in A and mCherry-F and GFP-F expressions were analyzed by confocal microscopy at 6 hpA in either intact (Ctrl) or amputated fins. Fin images are representative maximum projections of single fluorescence channels (mCherry-F, GFP-F) and overlay of fluorescences with transmitted light images (Merge). Arrowheads show mCherry-F^+^GFP-F^+^ cells and asterisk GFP-F^+^ cell, dotted lines outline the fin. Scale bar=20 *μ*m. (**c**) Number of *tnfa*^*+*^ and *tnfa*^−^ macrophages recruited to the wound region at indicated time points post amputation over 3 days. The number of larvae (*N*) used at each time point is indicated on the top of the graph. **P< 0.01 and ***P<0.001 indicated time point *versus* 0 hpA. Below the graph, a sketch of the different phases of recruitment of macrophage subsets: the M1-like macrophages accumulate during the early phase of regeneration (phase 1), whereas M2-like macrophages peaked at a later stage of the process (phase 2)

We previously described that caudal fin amputation in zebrafish larvae triggers macrophage activation and polarization and showed that *tnfa*^+^ macrophages express M1 markers *tnfb*, *il1b* and *il6* and *tnfa*^−^ macrophages express M2 markers *tgfb1, ccr2* and *cxcr4b.*^16^ To characterize more precisely macrophage behavior during caudal fin regeneration, we established the kinetic of macrophage subset recruitment after caudal fin amputation in 3 days post-fertilization (dpf) zebrafish. To specifically track macrophage subsets, we used the *Tg(mpeg1:mCherry-F/tnfa:eGFP-F)* double transgenic larvae, in which macrophages positive for *tnfa*, a marker of M1 macrophages, express both farnesylated forms of eGFP (GFP-F) and mCherry (mCherry-F)^16^ and imaged them at different times following amputation. In accordance with previous studies,^15, 21^ we observed that macrophages (*mpeg1*^+^) were rapidly recruited to the wound and remained present in the fin until complete regeneration at 3 days post amputation (dpA) (Figures 1a–c). *Tnfa*^+^ macrophages mainly accumulated at the wound from 6 h post amputation (hpA) with a peak at 6 hpA (Figures 1b and c). In contrast, *tnfa*^−^ macrophages were present in the injured fin from 6 hpA to 3 dpA (Figure 1c), with a peak between 24 and 72 hpA. This result suggests that two different subsets of macrophages undergo a sequential kinetic of recruitment during the regeneration process: the M1-like macrophages accumulated during the early phase of regeneration (referred as phase 1), whereas M2-like macrophages peaked at a later stage of the process (phase 2) (Figure 1c).

### Macrophage functions during caudal fin regeneration

**Figure 2:**
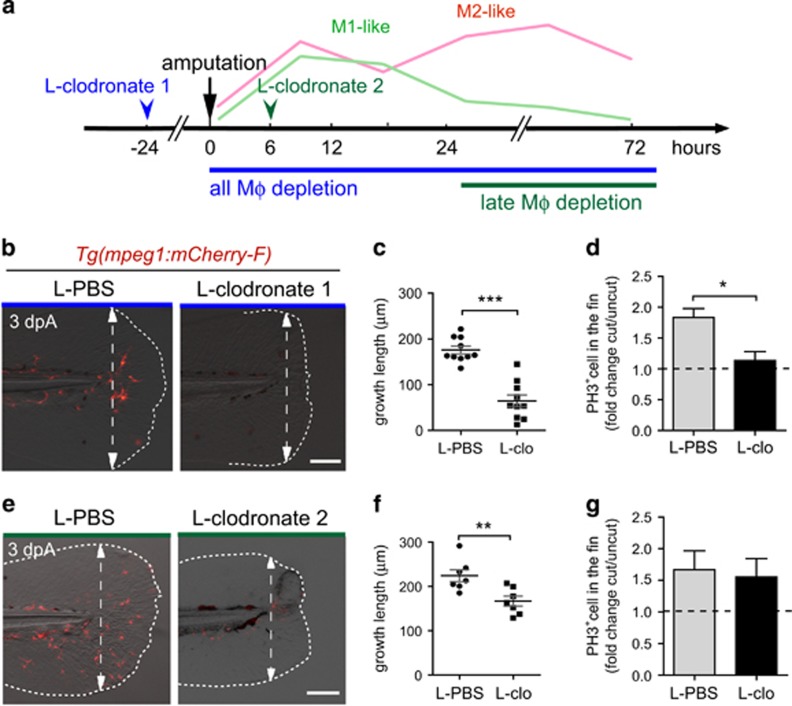
Macrophages are required for fin regeneration and blastemal cell proliferation in a stage-dependent manner. (**a**) Schedule of macrophage (M*ϕ*) depletion using L-clodronate injections. Macrophages were ablated using early L-clodronate injections (L-clodronate 1; no macrophages at the wound) or using injection of L-clodronate at later stages (L-clodronate 2; no more recruited M2-like macrophages from 24 hpA). (**b** and **e**) Consequences of macrophage depletion on caudal fin regeneration. (**b**) To deplete all macrophages from the early stage of regeneration, *Tg(mpeg1:mCherry-F)* were injected with L-clodronate (or L-clo) or L-PBS (control) at 48 hpf and fin were transected at 72 hpf (L-clodronate 1). (**e**) To deplete late-recruited macrophages, *Tg(mpeg1:mCherry-F)* were injected with L-clodronate or L-PBS at 6 hpA (L-clodronate 2). Fin images are representative overlays of mCherry fluorescence and transmitted light acquisitions at 3 dpA. Scale bar=100 *μ*m. Dotted lines outline the fin and dashed arrows, the position of the initial amputation. (**c** and **f**) Corresponding quantification of the regenerated fin length at 3 dpA after L-clodronate 1 treatment (**c**) and L-clodronate 2 treatment (**f**) in indicated conditions (mean±S.E.M., **P<0.01 and ***P<0.001). (**d**–**g**) Blastema cell proliferation at 24 hpA after L-clodronate 1 (**d**) and 2 (**g**) treatments in indicated conditions. Mitotic cells were detected using an anti-phosphorylated histone H3 (PH3) antibody (*N*_larvae_=10–15, average value of cut/uncut ratio±S.E.M, *P<0.05)

**Figure 3:**
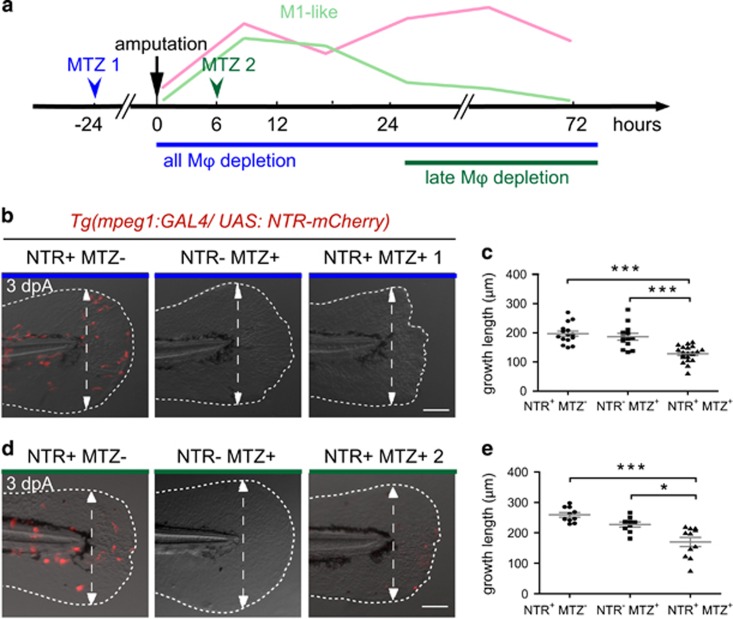
Genetic depletion of macrophages at different stages confirms the requirement of macrophages during fin regeneration. (**a**) Schedule of macrophage (M*ϕ*) depletion using *Tg(mpeg1:GAL4/UAS:NTR-mCherry)* larvae and Metronidazol (MTZ) treatment. (**b**–**e**) *Tg(mpeg1:GAL4/UAS:NTR-mCherry)* larvae were treated with MTZ (NTR^+^ MTZ^+^) to deplete macrophages. DMSO treatments on the same line (NTR^+^ MTZ^−^) or MTZ treatments on WT siblings (NTR^−^ MTZ^+^) were used as controls. (**b**) Treatments were performed from 48 hpf and fins were amputated at 3 dpf to deplete macrophages during the whole regeneration process (MTZ 1). (**d**) Treatments were performed from 6 hpA to deplete late macrophages (MTZ 2). Fin images are representative overlays of mCherry-F fluorescence (red) with transmitted light acquisitions at 3 dpA. Scale bar=100 *μ*m. Dotted lines outline the fin; dashed arrows indicate the position of the initial amputation. (**c** and **e**) Corresponding quantification of the regenerated fin length at 3 dpA in MTZ 1 (**c**) and MTZ 2 treatments (**e**) in indicated conditions (mean±S.E.M. from three independent experiments, ***P<0.001 and *P<0.05)

**Figure 4:**
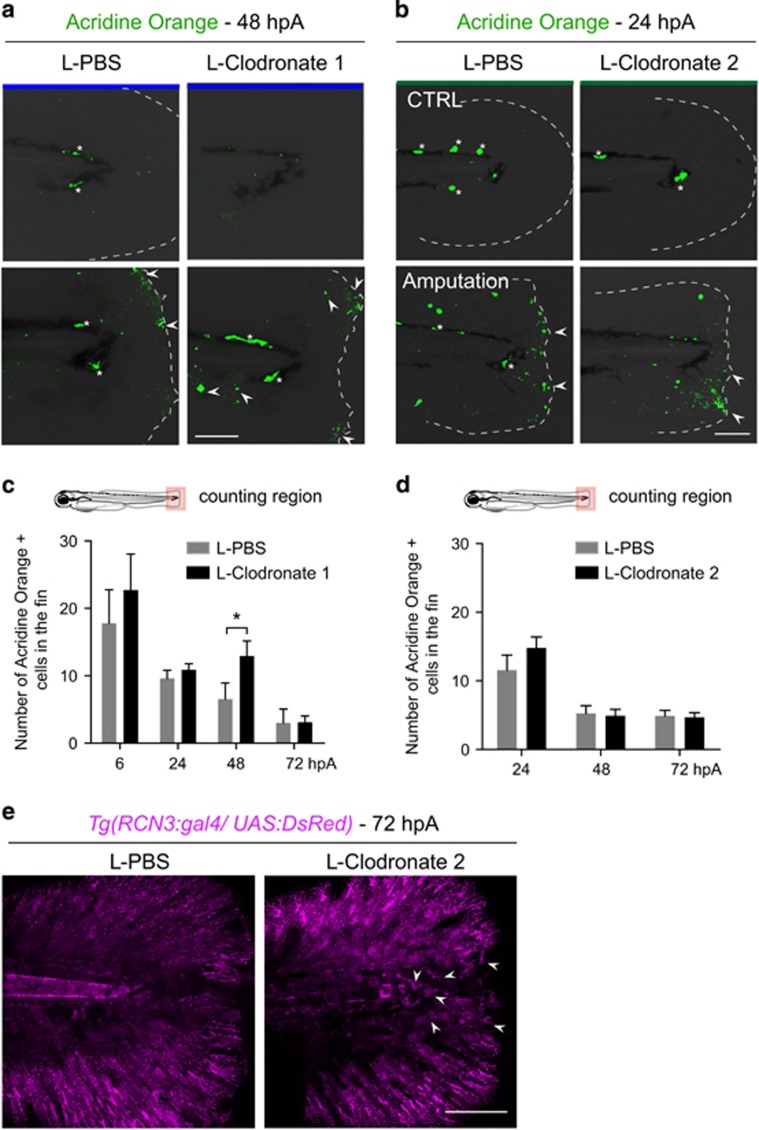
Depletion of late-recruited macrophages affects regeneration by impairing mesenchymal cell behavior but not cell death. (**a** and **b**) Representative images show cell death (green) in intact and amputated fin at (**a**) 48 hpA and (**b**) 24 hpA using confocal microscopy. (**a**) Cell death was detected using Acridine Orange staining in larvae in which all macrophages were depleted with L-clodronate treatment 24 h before amputation (L-clodronate 1) or not (L-PBS). (**b**) Cell death was detected using Acridine Orange staining in larvae in which late macrophages were depleted with L-clodronate treatment at 6 hpA (L-clodronate 2) or not (L-PBS). Dotted lines outline the fin, asterisks show autofluorescence of the pigments and arrowheads show dead cells. Scale bar=100 *μ*m. (**c**,**d**) Cell death counts in indicated conditions (*N*_larvae_=7–18 per group from two independent experiments, mean values±S.E.M., *P<0.05). (**e**) Tg*(rcn3:gal4/UAS:Ds-Red)* larvae were amputated at 3 dpf and injected with either L-PBS or L-clodronate at 6 hpA. Fin images are representative confocal maximum projections of Ds-Red (magenta) fluorescence in mesenchymal cells of the fins at 72 hpA. Arrowheads show round mesenchymal cells at the wound of late macrophage-depleted larvae. Scale bar=100 *μ*m

To determine the role of macrophages during the two phases of the regeneration process, we sequentially depleted macrophages. First, we used a chemical approach using liposome encapsulated clodronate that induces the death of phagocytic macrophages at two time points (L-clodronate 1 and L-clodronate 2) to target preferentially either pro- or anti-inflammatory macrophages (Figure 2a). Similar to a previous study,^22^ intravenous injection of L-clodronate in zebrafish larvae at 48 hpf efficiently lowered the total number of macrophages 24 h following injection, without affecting the number of neutrophils or inducing unspecific toxicity (Supplementary Figures 1a–c and data not shown). L-clodronate prevented macrophage recruitment to the wound following amputation (from 4 to 72 hpA) (Supplementary Figures 1d and e). This inhibition of M1- and M2-like macrophage recruitment after injection of L-clodronate 24 h before amputation impaired fin regenerative outgrowth, measured as length from the initial amputation position to the new distal fin edge (Figures 2b and c, L-clodronate 1). Epimorphic regeneration includes a boost of blastema cell proliferation as soon as 6 hpA in the region next to the stump of the larvae followed by the propagation of cell proliferation to more proximal regions from 24 hpA before being restored to a normal level at 3 dpA.^3^ Therefore, we assessed blastemal cell proliferation during the whole course of regeneration in L-clodronate injected larvae by means of immunodetection of phosphorylated histone 3 that labels proliferative cells (PH3^+^) and we reported a lower cell proliferation at the wound compared to controls that was significant at 24 hpA (Figure 2d; Supplementary Figure 2a). This was correlated with the downregulation of the blastemal marker *junbl*,^3^ as shown by *in situ* hybridization (Supplementary Figure 2b). This result reveals the pivotal role of macrophages during fin regeneration in zebrafish larvae, presumably by promoting cell proliferation and blastema formation. To examine the role of macrophages present in the injured fin in later stages (phase 2), L-clodronate was injected in *Tg(mpeg1:mCherry-F)* larvae at a time when pro-inflammatory *tnfa*^*+*^ macrophages are already recruited, at 6 hpA (L-clodronate 2, Figure 2a). A partial inhibition of macrophage recruitment was observed at 24 and 72 hpA and was correlated with both the impairment of fin outgrowth and morphological defects of the regenerated fin at 72 hpA (Figures 2e and f, L-clodronate 2 and not shown). By contrast to the early depletion of macrophages, the late depletion did not alter blastema cell proliferation (Figure 2g; Supplementary Figure 2c). Second, to further confirm the stage-dependent role of macrophages during regeneration, we used an alternate transgenic approach relying on the *mpeg1* promoter driving the expression of *E. coli* nitroreductase (NTR) enzyme to specifically deplete macrophages.^21, 22, 23^ Treatment with the pro-drug metronidazol (MTZ) at 48 hpf efficiently depleted macrophages at 3 dpf (Figure 3a; Supplementary Figures 3a and b). Similar to L-clodronate (L-clodornate-1) injection, MTZ treatment at 48 hpf (MTZ 1), 24 h before amputation, prevents macrophage recruitment from 6 hpA to 72 hpA (Supplementary Figures 3c and d) and altered the regeneration of the fin at 3 dpA (Figures 3b and c). Regeneration defects were associated with a lower expression level of *junbl* at the wound site and a reduced rate of blastema cell proliferation at 24 and 48 hpA (Supplementary Figures 3e and f). MTZ treatment at 6 hpA (MTZ 2) to deplete late-recruited macrophages also led to an impairment of the fin regeneration process (Figures 3d and e). However, the late macrophage depletion neither affects the proliferation of blastema cells nor *junbl* expression profile (Supplementary Figures 3g–h). To understand why the fin regeneration is altered in late macrophage-depleted larvae, we investigated cell death and the structure of the regenerating fins. We quantified dead cells in the fins of early and late macrophage-depleted larvae using Acridine Orange staining at different times following amputation. L-clodronate 1 and 2 treatments had no effect on cell death in intact fins (Figures 4a and b). Upon amputation, cell death was induced at the wound from 6 hpA. Although L-clodronate 1 treatment strengthened cell death at 48 hpA compared to control (Figures 4a and c), L-clodronate 2 treatment effect was similar to that of L-PBS control (Figures 4b and d). Mesenchymal cells, one of the main constituents of the fin fold at this stage, undergo marked changes of their shape during fin regeneration.^24^ To analyze the behavior of mesenchymal cells during regeneration, we amputated at 3 dpf the caudal fin from *Tg(rcn3:gal4/UAS:DsRed)* larvae, in which notochordal cells and mesenchymal cells of the fin are fluorescent. We observed that elongation of mesenchymal cells partially failed in larvae treated with L-clodronate 2 compared to controls (*N*_larvae_=17 for control and *N*_larvae_=5 for L-clodronate condition) (Figure 4e). This observation suggests that the ablation of late M2-like macrophages leads to regeneration defects by impairing mesenchymal cells behavior without modifying the rate of cell death or proliferation. Altogether, these results highlight the essential role of macrophages during fin regeneration, evidencing functionally distinct subpopulations. In addition, our data suggest a critical role of the early recruited macrophages in blastema formation. As M1-like macrophages mainly accumulate in the early phase (Figure 1c), we investigated the basis of the signal released by these macrophages in the orchestration of the regeneration.

### A role of TNF*α*/TNFR1 signaling in modulating fin regeneration

**Figure 5:**
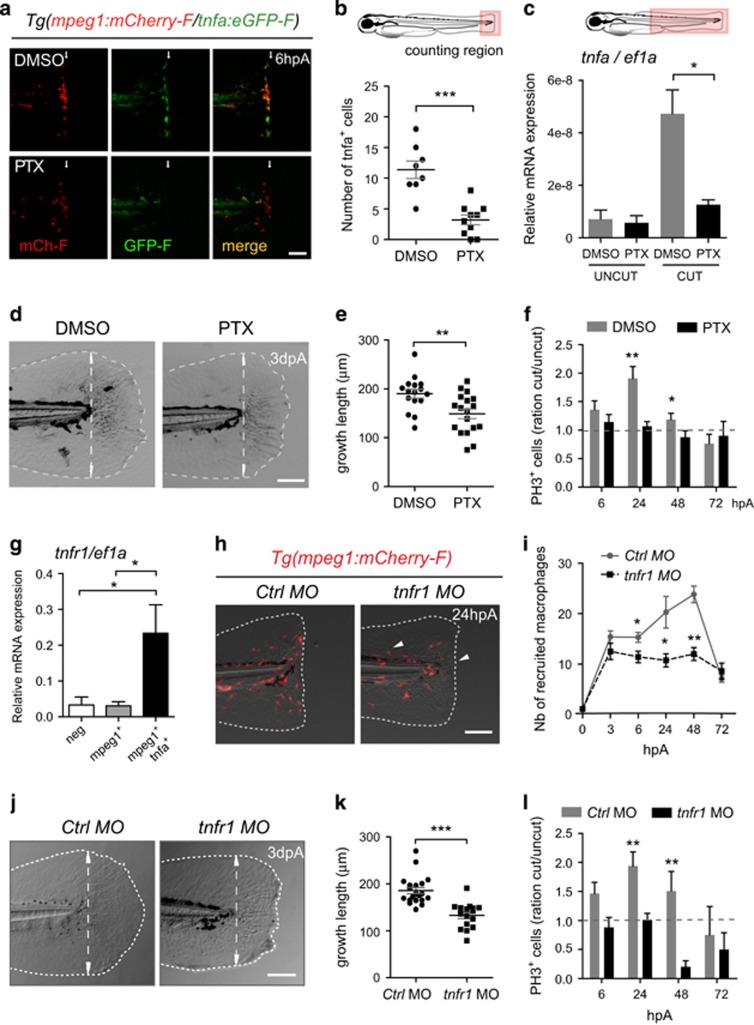
TNF signaling is required for caudal fin regeneration. (**a**) *Tg(mpeg1:mCherry-F/tnfa:eGFP-F)* larvae were amputated and treated with either DMSO or pentoxifylline (PTX). Representative fin images show single fluorescence channels and overlay of fluorescence (Merge) at 6 hpA. White arrows indicate the wound. (**b**) Number of GFP-F^+^ cells in the wound region in indicated conditions (*N*_larvae_=8–10, mean±S.E.M., two independent experiments, ***P<0.001). (**c**) qRT-PCR of *tnfa* mRNA relative to *ef1a* in DMSO or PTX treated larvae. Caudal fins were uncut or cut at 3 dpf and larvae were immediately treated with DMSO or PTX. RNA was extracted from tails (18 larvae per sample, mean±S.E.M. of three experiments, *P<0.05). (**d**) Representative transmitted light images of fins at 3 dpA after DMSO and PTX treatments. (**e**) Regenerated fin length at 3 dpA in indicated conditions (mean±S.E.M., three independent experiments, **P<0.005). (**f**) Blastema cell proliferation at 6, 24, 48 and 72 hpA in indicated conditions. Mitotic cells were detected in the fin region using an anti-PH3 antibody (*N*_larvae_=13–23, average of cut/uncut ratio±S.E.M., two independent experiments, *P<0.05, **P<0.005). (**g**) Steady state levels of *tnfr1* mRNA in sorted macrophages. *Tg(mpeg1:mCherry-F /tnfa:eGFP-F)* were amputated at 3 dpf and cells were collected at 6 hpA using fluorescence-activated flow cytometry and qRT-PCR used to quantify *tnfr1* mRNA steady state levels relative to *ef1a* in the following cell populations: mpeg1^−^tnfa^−^ (neg), mpeg1^+^tnfa^−^ (mpeg1^+^) and mpeg1^+^tnfa^+^ (mean values of four independent experiments±S.E.M. *P<0.05). (**h**) Fin images are representative overlays of mCherry fluorescence with transmitted light of *Tg(mpeg1:mCherry-F)* control morphants (*Ctrl* MO) or *tnfr1* morphants *(tnfr1* MO) at 24 hpA. (**i**) Macrophages (mCherry-F^+^) recruitment in the wound region at indicated time points in *Ctrl* and *tnfr1* morphants (*N*_larvae_=5–18 per group, mean±S.E.M., two independent experiments, *P<0.05, **P<0.005 respect to *Ctrl* MO condition). (**j**) Fin images are representative transmitted light images of *Ctrl* morphants and *tnfr1* morphants at 3 dpA. (**k**) Corresponding regenerated fin length (mean±S.E.M. from three independent experiments, ***P<0.001). (**l**) Blastema cell proliferation at 6, 24, 48 and 72 hpA in indicated conditions was detected using an anti-PH3 antibody (*N*_larvae_=5–17, cut/uncut ratio±S.E.M., two independent experiments, **P<0.01). (**a**, **d**, **h** and **j**) Dotted lines outline the fin, dashed arrows indicate the position of the initial amputation; scale bar=100*μ*m

TNF*α* is released by macrophages during the early stage of regeneration (Figures 1a–c and ref. 16), we decided to test TNF signaling involvement in regeneration using different inhibitory approaches. First, we used the pharmacological drug pentoxifylline (PTX) that was previously shown to inhibit efficiently tnfa transcription in various *in vitro* and *in vivo* systems.^25, 26, 27^ To evaluate the efficiency of the blocking in zebrafish larvae, *Tg(mpeg1:mCherry-F/tnfa:eGFP-F)* amputated larvae were treated with PTX. Compared to DMSO control, PTX treatment led to a decreased number of GFP-F^+^ cells in the fin at 6 hpA (Figures 5a and b), especially GFP-F^+^ macrophages and to a reduced number of recruited macrophages at the wound at 24 hpA (Supplementary Figures 4a and b). This was correlated with the decrease of *tnfa* mRNA expression in PTX treated fins at 5 hpA (Figure 5c). The effect of PTX treatment on other cytokines was monitored by measuring *il1b* and *il8* mRNA steady state levels: the amount of *il1b* mRNA was lowered while that of *il8* was not (Supplementary Figures 4c and d). This shows that PTX efficiently inhibits *tnfa* expression in zebrafish and also modulates *il1b* but to a lesser extent. Although PTX treatment did not affect the growth of intact fins (data not shown), it impaired the regenerative outgrowth of caudal fins at 3 dpA (Figures 5d and e). This was correlated with a decrease of the rate of blastema cell proliferation at 24 and 48 hpA compared to controls (Figure 5f) but no impairment of cell death (Supplementary Figure 4e). One of the *tnfa* receptors, *tnfrsf1a* (also known as *tnfr1*) was shown to be ubiquitously expressed in 3 dpf larvae.^28^ Using qRT-PCR on sorted cells at 6 hpA, we observed that *tnfr1* was expressed both by macrophages (mpeg1^+^ and mpeg1^+^ tnfa^+^) and non-macrophage cells (neg) although to a higher extent for M1-like macrophages (mpeg1^+^ tnfa^+^) (Figure 5g). To investigate more specifically the role of TNF*α*/TNFR1 signaling during fin regeneration, morpholino-mediated gene knockdown was used. First, using primers on either side of the splice sites targeted by the *tnfr1* morpholino *(tnfr1* MO), we provided evidence for the partially altered splicing patterns of *tnfr1* in 3 dpf *tnfr1* morphants (Supplementary Figure 5). Second, to examine the role of *tnfr1* in macrophage recruitment, we injected a *tnfr1* MO in *Tg(mpeg1:mCherry-F)* embryos. Although the overall morphology of *tnfr1* morphants without amputation as well as their total macrophage population were not significantly affected (data not shown), we observed that macrophages were recruited at 3 hpA. However their number at the wound was decreased as compared to control morphants (*Ctrl* MO) from 6 to 48 hpA (Figures 5h and i). This suggests that TNFR1 is not required for the initial recruitment of macrophages at the wound but acts to further enhance macrophage accumulation in the regenerating fin. At later stages (3 dpA), *tnfr1* morphants displayed partial defects of fin regeneration compared to control morphants (*Ctrl* MO) (Figures 5j and k). In parallel, *tnfr1* morphants displayed a significant decrease in blastema cell proliferation at 24 and 48 hpA as shown by PH3 immunodetection (Figure 5l). Similarly, fin regeneration was also impaired in *tnfa* morphants at 3 dpA (Supplementary Figures 6a and b), suggesting that these regeneration defects were specific and not because of the general morpholino-associated artifacts or off-target effects. Altogether, these results prove that the TNF*α*/TNFR1 signaling pathway is required for blastema cell proliferation and fin regeneration.

### Role of tnfr1 expressed by stromal cells during fin regeneration

**Figure 6:**
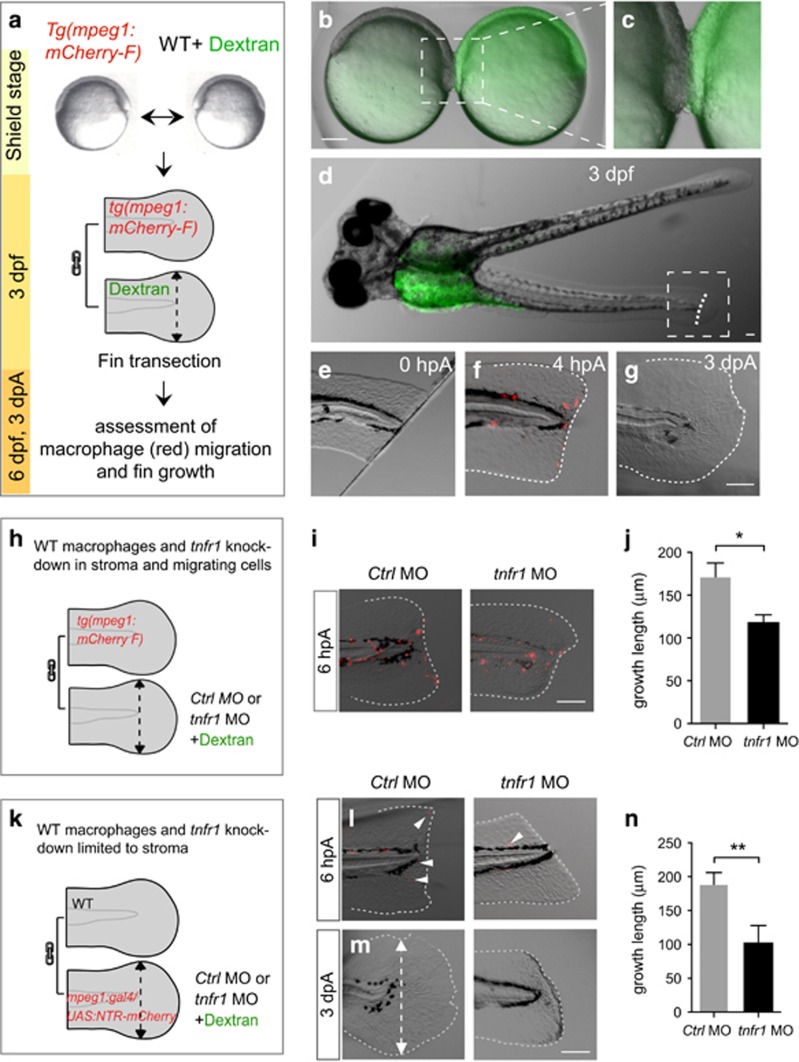
*tnfr1* knockdown in stromal cells impairs fin regeneration. (**a**–**g**) Macrophages migrate to the wound in parabiotic larvae following caudal fin amputation. (**a**) Schematic representation of the parabiosis experiment. (**b**) Generation of conjoined *Tg(mpeg1:mCherry-F)* and WT (Dextran-fluorescein) embryos at the shield stage, (**c**) high magnification of the region boxed in **b**. (**d**) At 3 dpf, conjoined larvae developed with rostral portion of the body fused. WT larva was identified using the green fluorescence of dextran-fluorescein in the yolk sac and selected for caudal fin amputation (dashed line). (**e**) The caudal fin from WT larva was amputated at 3 dpf, 0 hpA; no mCherry-F^+^ macrophages were detected in the fin. (**f**) After amputation mCherry-F^+^ macrophages migrate to the wound at 4 hpA and (**g**) the fin completely regenerate at 3 dpA (*N*_larvae_=6, scale bars=100 *μ*m). (**h**) Schematic representation of the parabiosis experiment using *Tg(mpeg1:mCherry-F)* and *tnfr1* (*tnfr1* MO) or *control* (*Ctrl* MO) morphants. Caudal fin of morphant was amputated at 3 dpf in the region indicated with dashed arrow. (**i**) Representative fin images of mCherry fluorescence merged with transmitted channel at 6 hpA show *tnfr1*^*WT*^ macrophages (mCherry-F^+^) recruited in the amputated caudal fin of both *tnfr1* and *ctrl* morphants. (**j**) Corresponding quantification of the regenerated fin length at 3 dpA in indicated conditions (*tnfr1 MO*, *N*_larvae_=6 and *Ctrl MO, N*_larvae_=8. (**k**) Schematic representation of the parabiosis experiment using WT and *Tg(mpeg1:GAL4/UAS:NTR-mCherry)* that were previously injected with *tnfr1* MO or *Ctrl* MO. To induce macrophage depletion in one of the partner, parabiotic larvae were treated with MTZ at 48 hpf and the caudal fin of the morphant (fluorescein) was amputated at 3 dpf. (**l**) Representative fin images of mCherry fluorescence merged with transmitted channel at 6 hpA show NTR-mCherry^+^ macrophages that are mainly depleted. White arrowheads show residual fluorescence in cells or cell fragments. (**m**) Fin images are representative transmitted light images at 3 dpA in *tnfr1* and *Ctrl* morphants. (**n**) Corresponding quantification of the regenerated fin length in indicated conditions (*N*_larvae_=4–6 mean±S.E.M., *P<0.05). (**f**, g, **i**, **l** and **m**) Dotted lines outline the fin. (**m**) Dashed arrows indicate the position of the initial transection. Scale bars=100 *μ*m

We then hypothesized that TNF*α* producing macrophages promote regeneration through direct activation of the proliferation of blastemal stromal cells mediated by TNFR1. To address that hypothesis and accurately dissect the function of *tnfr1* gene in migrating cells *versus* stromal cells, we performed parabiosis experiments to surgically generate conjoined zebrafish embryos sharing a common bloodstream.^29^ To test whether macrophages can migrate from one partner of the parabiote pair to the other, we fused a transgenic *Tg(mpeg1:mCherry-F)* gastrula with a green fluorescent wild-type (WT) gastrula (Figures 6a–c). At 3 dpf, fused embryos displayed separated trunk and tail (Figures 6a and d) and mCherry-F^+^ cells have invaded the WT parabiont (data not shown). The WT parabiont was discriminated using fluorescein and its caudal fin was amputated at 3 dpf (Figures 6b and d). At 0 hpA, no mCherry-F^+^ macrophages were present at the wound but at 3 hpA, mCherry-F^+^ macrophages originating from the transgenic parabiont migrate to the wound (Figures 6e and f). At 3 dpA, the caudal fin was correctly regenerated, demonstrating the relevance of the chosen approach for regenerative studies (Figure 6g).

To determine whether *tnfr1* was required on blastemal stromal cells for fin regeneration, we performed similar parabiosis experiments combined with morpholino-mediated silencing approach. Fused *Tg(mpeg1:mCherry-F)* embryos and green fluorescent *tnfr1* morphants were generated, and the caudal fin of the morphant was amputated at 3 dpf to test the migratory potential of *mCherry-F*^+^ *tnfr1*^*WT*^ macrophages in morphants. We observed that *mCherry-F*^+^ *tnfr1*^*WT*^macrophages were similarly recruited to the wound in *Ctrl* and *tnfr1* morphants at 6 hpA (Figures 6h and i). At 3 dpA, whereas *Ctrl* morphant partner regenerated, *tnfr1* morphant partner failed to regenerate properly (Figure 6j). To limit *tnfr1* knockdown to stromal cells, we injected the *tnfr1* MO in *Tg(mpeg1:GAL4/UAS:NTR-mCherry)* embryo and gastrulae from these morphants were then fused to WT gastrulae (Figure 6k). MTZ treatment at 48 hpf resulted in the death of most *NTR-mCherry*^*+*^ macrophages at 3 dpf. Then, after amputation of the caudal fin of the morphants, we observed very few *NTR-mCherry*^*+*^ macrophages at the wound (Figure 6l). Although *NTR-mCherry*^*+*^ macrophages were depleted, regeneration occurred normally in *Ctrl* morphants at 3 dpA (Figure 6m). These results show that parabiosis restored the regeneration potential in the *Tg(mpeg1:GAL4/UAS:NTR-mCherry) Ctrl* morphants, presumably through the recruitment of macrophages originating from the *WT* parabiont. Conversely, the mobilization of *tnfr1* competent macrophages did not rescue the regeneration potential of the fins in *tnfr1* morphant in which stromal cells were silenced for *tnfr1* (Figures 6m and n). These results show that *tnfr1* is expressed by stromal cells and mediates *tnfa*-dependent caudal fin regeneration.

Finally, to determine whether macrophages lacking *tnfa* can stimulate regeneration, we performed similar parabiosis experiments with *tnfa* morphants as a macrophage donors and MTZ-treated *Tg(mpeg1:GAL4/UAS:NTR-mCherry)* embryos as wounded parabionts (Supplementary Figure 6c). At 6 hpA, most NTR-mCherry^+^ macrophages were depleted by MTZ treatment (Supplementary Figure 6d), and at 3 dpA, parabiosis which received the *tnfa* MO regenerate less than the controls (Supplementary Figures 6c–f), suggesting that macrophages lacking *tnfa* are less efficient in stimulating regeneration.

## Discussion

**Figure 7:**
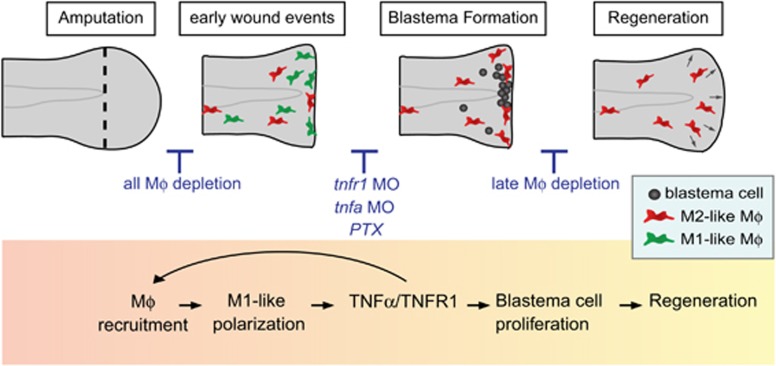
Model of macrophage recruitment and TNF*α* signaling during the initiation of blastema formation and caudal fin regeneration. Caudal fin amputation in 3 dpf zebrafish larva leads to an early inflammatory response characterized by an accumulation of M1-like macrophages at the wound site from 6 to 24 hpA followed by an accretion of M2-like macrophages. This tightly regulated M1/M2 balance controls the different stages of the regeneration process. Indeed, M1-like macrophages expressing *tnfa* activates the TNF*α*/TNFR1 axis to enhance macrophage recruitment to the wound. Furthermore, M1-like macrophages-mediated TNF*α* controls blastema cell proliferation through the receptor TNFR1. Late M2-like macrophages participate to fin remodeling

Although much effort has been made to understand the basis of epimorphic regeneration, the molecular mechanisms by which macrophages mediate signaling are poorly documented. Here, we demonstrate a novel role for macrophage-derived TNF signaling in orchestrating the regeneration process in response to fin injury in zebrafish (Figure 7).

In this study we identify TNF*α* as one of the key signals expressed transiently by polarized macrophages during early phases of regeneration, that control regeneration process. First, we showed that treatment with PTX, a non-selective phosphodiesterase inhibitor that efficiently inhibits TNF*α* responses *in vitro* and *in vivo*, impairs regeneration. Although PTX inhibits *tnfa* mRNA production in zebrafish without markedly affecting other pro-inflammatory cytokines, *tnfa* may not be the only target of PTX. Second, using morpholinos targeting both the receptor *tnfr1* and the ligand *tnfa*, we showed that TNFR1/TNF*α* axis is necessary for proper regeneration. In our system TNF*α* promotes macrophage recruitment, presumably acting as an enhancing loop for macrophage accumulation to the wound (Figure 7). TNF*α* is unlikely the first signal that triggers macrophage recruitment, as TNF signaling knockdown did not affect the initial migration of macrophages to the injured fin nor abolish completely their recruitment. Other chemoattractant molecules, including the contents of damaged cells, chemokines produced by intact cells and a burst of H_2_O_2_, are known to be released by the wound and trigger leukocytes recruitment.^30^ These molecules may acts as primary a signal for macrophage recruitment independently of *tnfa*. The second role of TNF*α* signal is to promote regeneration by stimulating proliferation of blastemal cells. Parabiosis experiments combined with morpholino knockdown strategy strongly suggest that TNF*α* acts directly on stromal cells through its receptor TNFR1 (Figure 7). Indeed, parabiosis is able to rescue fin regeneration in macrophage-depleted larvae that are fully competent for *tnfr1.* By contrast, no circulating cells, including macrophages, nor soluble factors can restore regenerative potential in *tnfr1* incompetent fins. Furthermore, we show that macrophages lacking *tnfa* are less efficient in stimulating regeneration in parabiosis. Although the effects of TNF*α* can be very versatile, our results are in line with studies suggesting its role in retinal regeneration by promoting the proliferation of progenitor cells.^31, 32, 33^ The necessary role of TNF signaling in fin regeneration suggests that self-limiting acute inflammation is essential for a proper regenerative response. Petri and collaborators previously showed that Wnt/*β*-catenin signaling is crucial for blastema formation and regenerative outgrowth of the fin in adult zebrafish, regulating macrophage recruitment and cytokine secretion.^15^ Wnt/*β*-catenin might be an upstream signal of TNFa/TNFR1 that controls the progression of regeneration by regulating the balance of macrophage phenotypes.

Evidences are emerging that regeneration relies on the timely controlled engagement of immune cell at the wound site. Although macrophages are required for limb regeneration in adult Axolotl^10^ and for caudal fin regeneration in adult zebrafish,^15^ their role in fin regeneration during larval stages has been controversial.^7, 12, 13, 14^ Using two independent approaches, we efficiently and specifically ablated macrophages at different time points during the multi-stage regeneration process of the larvae fin. Both chemical and genetic ablation of macrophages show that macrophages, recruited in the early phase, including M1-like and M2-like, are required for the fin regeneration through the stimulation of blastemal cell proliferation. By contrast, macrophages that are present during the late phase of regeneration and arbor an M2-like phenotype may promote regeneration by remodeling mesenchymal cells. These results are in agreement with what has been observed in adult zebrafish and salamander^10, 15^ and emphasize the similarity between adult and larval regeneration mechanisms. Although M2 polarized macrophages are thought to be preferentially associated with tissue repair, our results suggest that M1-like macrophages, through the production of TNF*α*, create a permissive environment preparing the ground for efficient regeneration. The positive role of M1-like macrophages, as trophic factor producers, is reminiscent of what has been observed during skeletal muscle regeneration after injury in mammals where blood derived monocyte/macrophages with M1 state stimulate the proliferation of myogenic precursor cells through the secretion of molecules like IL-6, TNF*α* and IL1-*β*.^6, 34, 35^ In mammals, M2 macrophages do not constitute a uniform population and often are further subdivided into M2a, M2b and M2c categories.^36, 37^ Similar to mammals, M2-like macrophages from zebrafish larvae may encompass subgroups exerting diverse functions during regeneration. Indeed early M2-like might not have the same function as late M2-like macrophages. Recently, macrophages were shown to attenuate *il1-b* expression in the wound epithelium, preventing excessive cell death in the regenerative fin.^38^ As previously shown, early M2-like macrophages express anti-inflammatory genes like *tgf-β1*,^16^ suggesting they are responsible for decreasing *il1-b*-mediated inflammation. In our study late M2-like macrophages have no role on cell proliferation and cell death but instead control mesenchymal cell behavior during later phases of regeneration. Whether that function is mediated by direct contact or through the expression of trophic factors is still unknown.

In conclusion, we propose that the epimorphic regeneration process depends on a narrow window of action of TNF*α* expressed by M1-like macrophages activated upon caudal fin amputation. The critical effect of TNF*α* on proliferation and the fate of blastema cells depends on TNFR1.

## Materials and methods

### Ethics statement

All animal experiments described in the present study were conducted at the University of Montpellier according to European Union guidelines for handling of laboratory animals (http://ec.europa.eu/environment/chemicals/lab_animals/home_en.htm) and were approved by the Direction Sanitaire et Vétérinaire de l'Hérault and Comité d'Ethique pour l'Expérimentation Animale under references CEEA-LR-13007 and 2016061511212601.

### Zebrafish line and maintenance

Fish and embryo maintenance, staging and husbandry were as previously described.^39^ Experiments were performed using the AB zebrafish stain (ZIRC), using the transgenic line *Tg(mpeg1:mCherry-F)*^*ump2Tg*^ to visualize macrophages,^21, 39^ *Tg(tnfa:eGFP-F)*^*ump5Tg*^ to visualize *tnfa* expression,^16^ *Tg(mpx:eGFP)*^*i114*^ to visualize neutrophils,^40^ *Tg(rcn3:gal4/UAS:DsRed)* (PD1023) (PD1112) to visualize mesenchymal cells,^41^ *Tg(mpeg1:Gal4FF)* (here cited as *Tg(mpeg:Gal4*))^21^ and *Tg(UAS-E1b:Eco.NfsB-mCherry)* (here cited as *Tg(UAS:NTR-mCherry))*^42^ to genetically deplete macrophages. Embryos were obtained from pairs of adult fish by natural spawning and raised at 28.5 °C in tank water. Embryos and larvae were staged according to Kimmel *et al.*^43^

### Larva manipulation for regeneration assays and imaging

Caudal fin amputation was performed on 3 dpf larvae as described in ref. 4,4. The caudal fin was amputated with a sterile scalpel, posterior to muscle and notochord under anesthesia with 0.016% Tricaine (ethyl 3-aminobenzoate, Sigma-Aldrich, France) in zebrafish water. For imaging, larvae were anesthetized in 0.016% Tricaine, positioned in 35 mm glass-bottom dishes (FluroDish, World Precision Instruments, UK), immobilized in 0.8% low melting point agarose (Sigma) and covered with 2 ml of embryo water containing tricaine. Epi-fluorescence microscopy was performed using a MVX10 Olympus microscope equipped with MVPLAPO × 1 objective and XC50 camera. Confocal microscopy was performed using an inverted confocal microscope TCSSP5 SP5 with a HCXPL APO × 40/1.25–0.75 oil and a HC PL APO 0.70 ∞(infinity) × 20 objective (Leica Microsystems, France). The 3D files generated by multi-scan acquisitions were processed using Image J (NIH).

### Morpholino injections

For *tnfr1* (also known as *tnfrsf1a,* NM_213190) loss-of-function experiments, we used morpholino antisense oligonucleotides (Gene Tools, Philomath, OR, USA) – that specifically hybridized with Exon 5-Intron 5 splicing site (MO zTNFRsplD5 referred here as *tnfr1 MO*): 5′-GGAAGCATGAGGAACTTACAGTTCT-3′. For *tnfa* loss-of-function experiments, *tnfa MO* (MO tnfaD1) that specifically hybridized with Exon 1- Intron 1 splicing site was used (5′-GGGCAGGATTTTCACCTTATGGAGC-3′). As a control, Control morpholino from Gene Tools was used (ctrl MO, 5′-AATCACAAGCAGTGCAAGCATGATG-3′).

7 ng of *tnfr1*, *tnfa* or control morpholinos were injected in one-cell stage embryos with a Femto.Jet from Eppendorf. No side effect was observed. Efficiency was tested by RT-PCR, using primers from both sides of the morpholino target: zTNFR1.5 (5′-CAGGAATGCAGTGCAGAAAA-3′) and zTNFR1.30 (5′-AAAAAGACTGGGGGAATGCT-3′).

### Macrophage ablation and drug treatments

For macrophage ablation using L-clodronate injection, the larvae were anesthetized in 0.016% Tricaine and microinjected with 5 nl of liposome encapsulated clodronate (www.clodronateliposomes.org) in the posterior caudal vein in the Urogenital Opening region at 48 hpf or 3 dpf. Control embryos were similarly injected with liposome-PBS (L-PBS). For macrophage ablation experiments using Metronidazol, the double transgenic larvae *Tg(mpeg:Gal4; UAS:NTR-mCherry),* were incubated in zebrafish water supplemented with or without 5 mM metronidazol (MTZ, Sigma-Aldrich), either 24 h before amputation or at 6 hpA for the duration of the experiment. Larvae were maintained in the dark as MTZ is sensitive to long light exposure. As controls, NTR transgenic larvae were incubated in fish water without MTZ, and wild-type siblings were incubated in zebrafish water with MTZ (5 mM) under the same conditions. Ablation efficiency was assessed by imaging using the MVX10 Olympus microscope (MVPLAPO × 1 objective and XC50 camera, Olympus, France), and by cell counts using FACS LSRFortessa (BD Biosciences, France). Cytometry data were analyzed using the Flowjo software (Tree start, Ashland, OR, USA). For *tnfa* transcriptional inhibition, larvae were incubated in zebrafish water supplemented with either 0.35% DMSO (control) or with 35 *μ*M of Pentoxifylline (Sigma-Aldrich) immediately after caudal fin amputation at 3 dpf.

### Parabiosis experiments

Generation of parabiotic embryos was as previously^29^ with the following modifications. Morpholinos and dextran were injected at one-cell stage. Embryos were manually dechorionated at the 256-cell stage with fine forceps in two separate glass Petri dishes in low-calcium Ringer’s solution (5 M NaCl solution, 3 M KCl solution and 1 M HEPES) containing antibiotics (50 U/ml penicillin–streptomycin). Two partner embryos, one of each of the two genetic backgrounds to be fused were then transferred into 2 mm diameter agarose wells filled with high-calcium Ringer’s solution (low-calcium Ringer’s solution complemented with 5 mM CaCl_2_. A 25G needle was then used to detach a few cells from both shield regions at their contact point. Embryos were pushed to press the wounds against each other. After shield fusion, embryos were incubated for 20–30 min in the same buffer. High-calcium Ringer’s solution was then replaced by low-calcium Ringer’s solution complemented with antibiotic and embryos were then maintained at 28 °C to develop. Ringer solution was replaced by embryo water at 24 hpf.

### FACS purification, mRNA isolation from macrophage subsets and qRT-PCR on sorted cells

mCherry-F^+^GFP-F^−^ and mCherry-F^+^GFP-F^+^ cell sorting from 300 *Tg(mpeg1:mCherry-F/tnfa:eGFP-F)* larvae following amputation, mRNA extraction and qRT-PCR were performed as previously described,^16^ and *tnfr1* expression was detected using zTNFR1.5 (see above) and zTNFR1.3 (5′-CGAGAGCATTCCCATCCTAA-3′).

### RNA preparation on larva tails and quantitative RT-PCR

To determine the relative expression of *tnfa*, *il1b* and *il8*, total RNA from larva tails (pools of 10 or 18 tails each) was prepared at different time points post amputation. RNA preparation and reverse transcription were as described in ref. 4,5. QRT-PCR analyses were performed using LC480 software. The primers used were the following: zTNFa.54 (5′-TTCACGCTCCATAAGACCCA-3′), zTNFa.34 (5′-CCGTAGGATTCAGAAAAGCG-3′), zIl1b.5 (5′-TGGACTTCGCAGCACAAAATG-3′), zIl1b.3 (5′-GTTCACTTCACGCTCTTGGATG-3′), zIl8.5 (5′-CCTGGCATTTCTGACCATCAT-3′), zIl8.3 (5′-GATCTCCTGTCCAGTTGTCAT-3′), zef1a.5 (5′-TTCTGTTACCTGGCAAAGGG-3′), zef1a.3 (5′-TTCAGTTTGTCCAACACCCA-3′).

### *In situ* hybridization, proliferation detection and dead cell detection

Plasmid-containing junb-l (PCRII-junb-l), was kindly sent by Atsushi Kawakami (Department of Biological Information, Tokyo Institute of Technology). Digoxigenin (DIG)-labeled (Roche, France) sense and antisense RNA probes were obtained by *in vitro* Transcription (Biolabs, France). *In situ* hybridizations on whole-mount embryos were performed as previously described.^46^ Stained embryos were imaged using a MVX10 Olympus microscope (MVPLAPO × 1 objective and XC50 camera) and using a Zeiss Axioimager (Zeiss × 40 Plan-Apo 1.3 oil objective) (Zeiss, France). For quantification of cell proliferation, whole embryos were fixed in paraformaldehyde 4% and stained as described in ref. 46 using an anti-phosphorylated histone 3 antibody (Cell Signaling, France, ref 9701, 1/500) and a secondary antibody Goat anti-Rabbit coupled with Alexa Fluor 488 (A-11034, Life Technologies-Invitrogen, France). For cell death quantification, the embryos were placed in Petri dishes containing 5 *μ*g/ml of Acridine orange (Sigma-Aldrich, France) diluted in zebrafish water from a 10 mg/ml stock solution and incubated during 30 min. Embryos were then washed with zebrafish water three times 10 min each. The embryos were then replaced at 28 °C until observation by confocal microscopy.

### Monitoring of fin regeneration, macrophage subsets count and statistical analysis

Caudal fin regeneration was monitored by measuring the length of fin growth from the transected plan (end of the notochord) up to the most proximal end of the fin. Macrophages and cell proliferation and cell death in the wound region were counted directly on images acquired by microscopy using specific reporter lines and stainings (see fish lines and cell proliferation detection and death sections). Graphs show mean±standard error of the mean (S.E.M.). Mann–Whitney, two tails was performed to test significance for Figures 1c, 2c, d, f and g, and 5b, e, i, k and l; and Supplementary Figures S1b, S1e, S2a, S2c, S3b, S3d, S3f, S3h, S4a, S4b, and S6b using GraphPad Prism 5 Software (San Diego, CA, USA). Mann–Whitney, one tail was performed to test significance for Figures 4c and d,5c, f and g,6j and n and Supplementary Figures S4c, S4d, S4e and S6f. ANOVA using Kruskal–Wallis with a Dunn’s post-test was performed to test significance for Figures 3c and e using GraphPad Prism 5 Software.
