# Correction to: TNF signaling and macrophages govern fin regeneration in zebrafish larvae

**DOI:** 10.1038/s41419-018-0751-2

**Published:** 2018-06-13

**Authors:** Mai Nguyen-Chi, Béryl Laplace-Builhé, Jana Travnickova, Patricia Luz-Crawford, Gautier Tejedor, Georges Lutfalla, Karima Kissa, Christian Jorgensen, Farida Djouad

**Affiliations:** 1 IRMB, INSERM, University of Montpellier, Montpellier, France; 2 DIMNP, CNRS, University of Montpellier, Montpellier, France; 3 Laboratorio de Immunologia Celular y Molecular, Facultad de Medicina, Universidad de Los Andes, Santiago, Chile; 4 Clinical Unit for Osteoarticular Diseases and Department for Biotherapy, CHU Lapeyronie, Montpellier, France

**Correction to**: *Cell Death Dis*. **8**, e2979 (2017); 10.1038/cddis.2017.374; published online 10th August 2017

Following publication of the article, the authors spotted errors in the affiliations and footnote on page 1. They should read as follows:

Mai Nguyen-Chi*^,1,2,6^, Béryl Laplace-Builhé^1,6^, Jana Travnickova^2^, Patricia Luz-Crawford^1,3^, Gautier Tejedor^1^, Georges Lutfalla^2^, Karima Kissa^2^, Christian Jorgensen^1,4,5^ and Farida Djouad^*,1,5^

^1^IRMB, INSERM, University of Montpellier, Montpellier, France; ^2^DIMNP, CNRS, University of Montpellier, Montpellier, France; ^3^Laboratorio de Immunologia Celular y Molecular, Facultad de Medicina, Universidad de Los Andes, Santiago, Chile; ^4^Clinical Unit for Osteoarticular Diseases and Department for Biotherapy, CHU Lapeyronie, Montpellier, France. ^5,6^These authors contributed equally to this work. * Correspondence to Mai Nguyen-Chi (mai.nguyen-chi@univ-montp2.fr) and Farida Djouad (farida.djouad@inserm.fr)

