# Supplementary material for: TNF signaling and macrophages govern fin regeneration in zebrafish larvae
Source: Cell Death Dis. 2017 Aug 10;8(8):e2979–. doi: 10.1038/cddis.2017.374 (PMC5596562; doi:10.1038/cddis.2017.374)
Supplement: Supplementary Information [file cddis2017374x1.pdf]

Supplementary Information

TNF signalling and macrophages govern fin regeneration in zebrafish larvae

Running title: TNF signalling in fin regeneration in zebrafish

Authors

Mai Nguyen-Chi[1,2,3,6*], Béryl Laplace-Builhé[1,2,6], Jana Travnickova[2,3], Patricia Luz-Crawford[1,2,5], Gautier Tejedor[1,2], Georges Lutfalla G[3], Karima Kissa [3], Christian Jorgensen[1,2,4,7], Farida Djouad[1,2,7*]

Figure S1

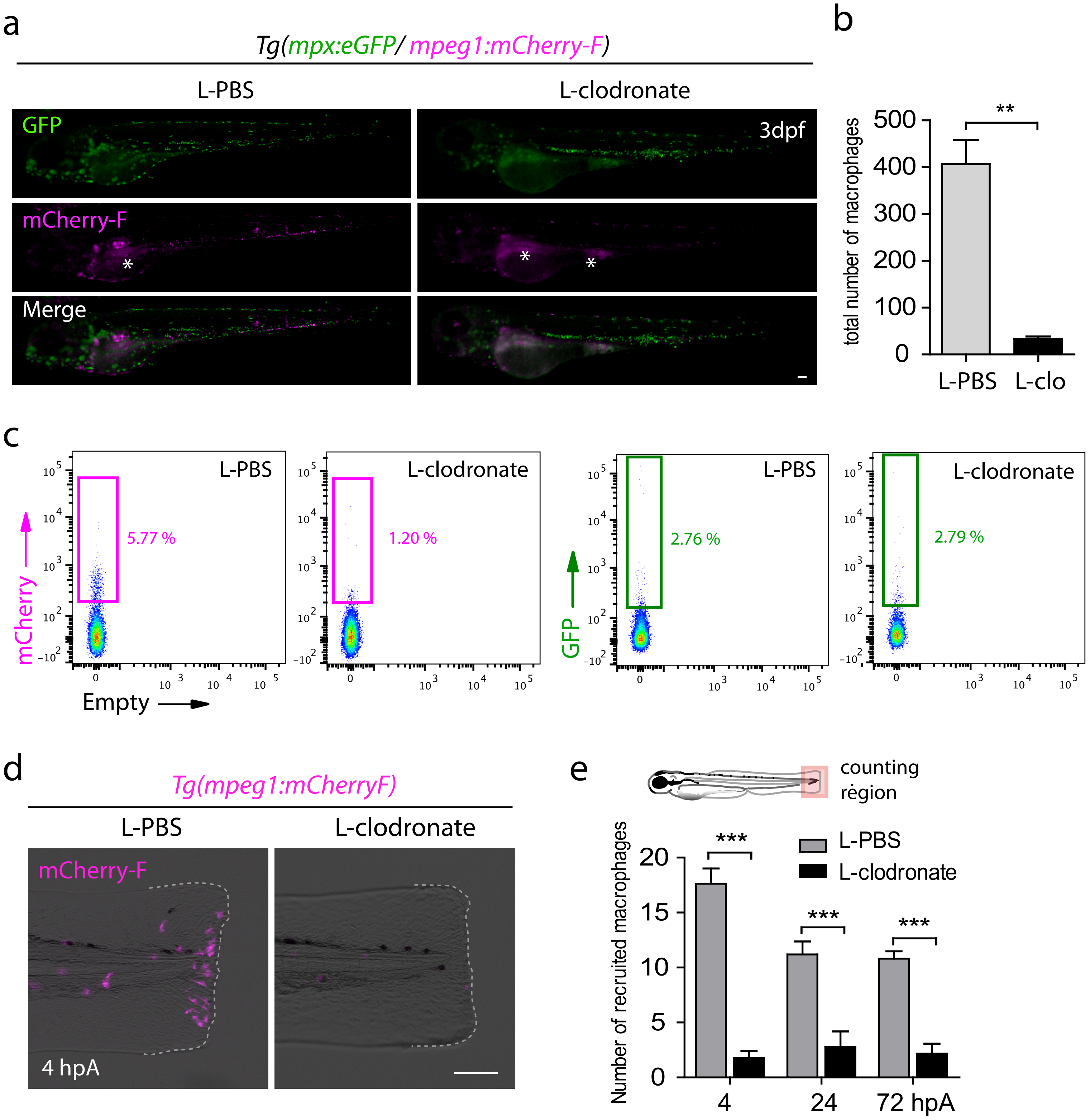

a
Tg(mpx:eGFP/ mpeg1:mCherry-F)
L-PBS
L-clodronate
GFP
3dpf
mCherry-F
Merge
b
total number of macrophages
**
500
400
300
200
100
0
L-PBS
L-clo
c
L-PBS
L-clodronate
5.77 %
1.20 %
2.76 %
2.79 %
mCherry
GFP
Empty
d
Tg(mpeg1:mCherryF)
L-PBS
L-clodronate
mCherry-F
4 hpA
e
counting region
Number of recruited macrophages
***
L-PBS
L-clodronate
20
15
10
5
0
4
24
72 hpA

Figure S2

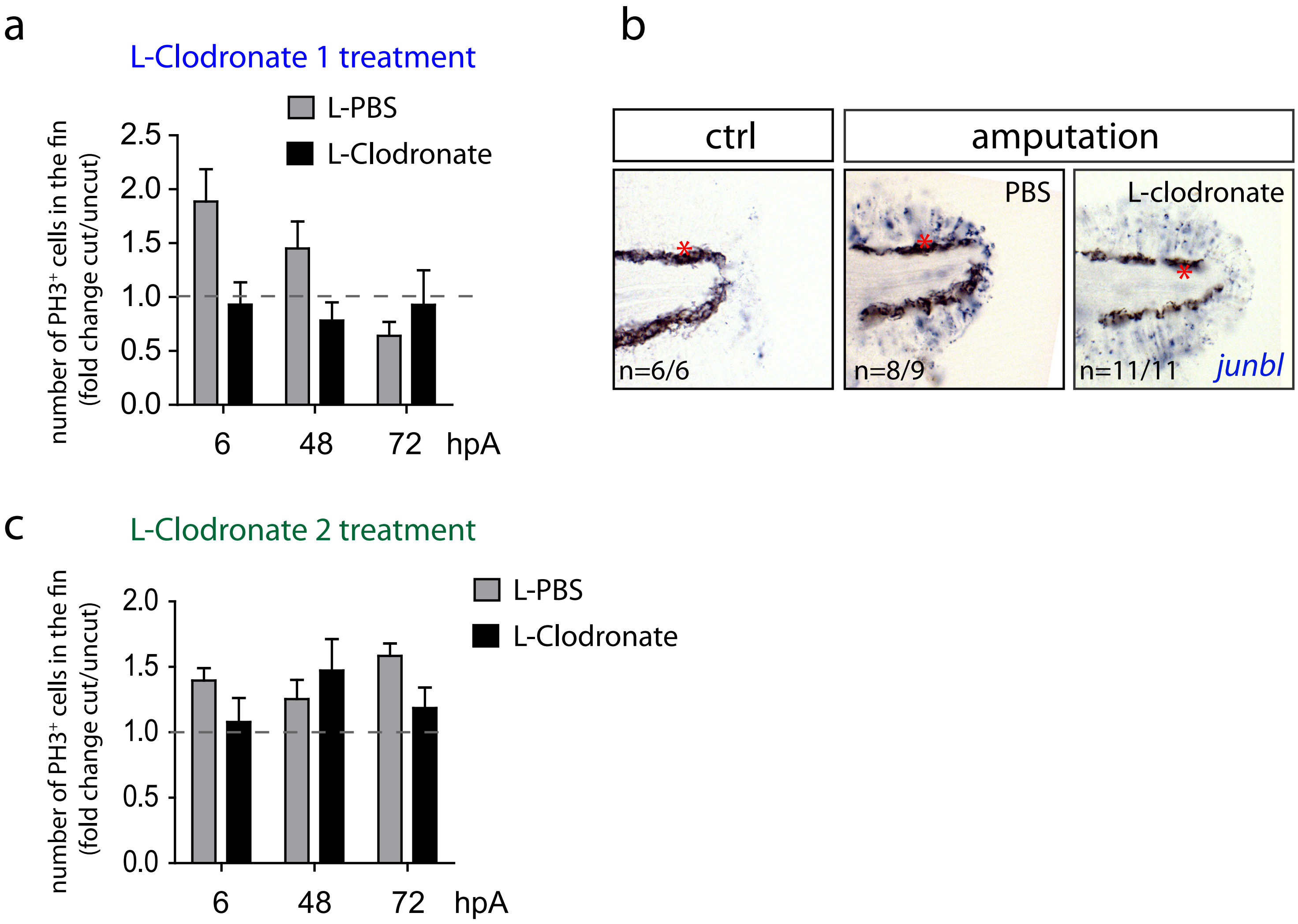
a
L-Clodronate 1 treatment
L-PBS
L-Clodronate
number of PH3+ cells in the fin (fold change cut/uncut)
2.5
2.0
1.5
1.0
0.5
0.0
6
48
72
hpA
b
ctrl
amputation
PBS
L-clodronate
n=6/6
n=8/9
n=11/11
junbl
c
L-Clodronate 2 treatment
L-PBS
L-Clodronate
number of PH3+ cells in the fin (fold change cut/uncut)
2.0
1.5
1.0
0.5
0.0
6
48
72
hpA

Figure S3

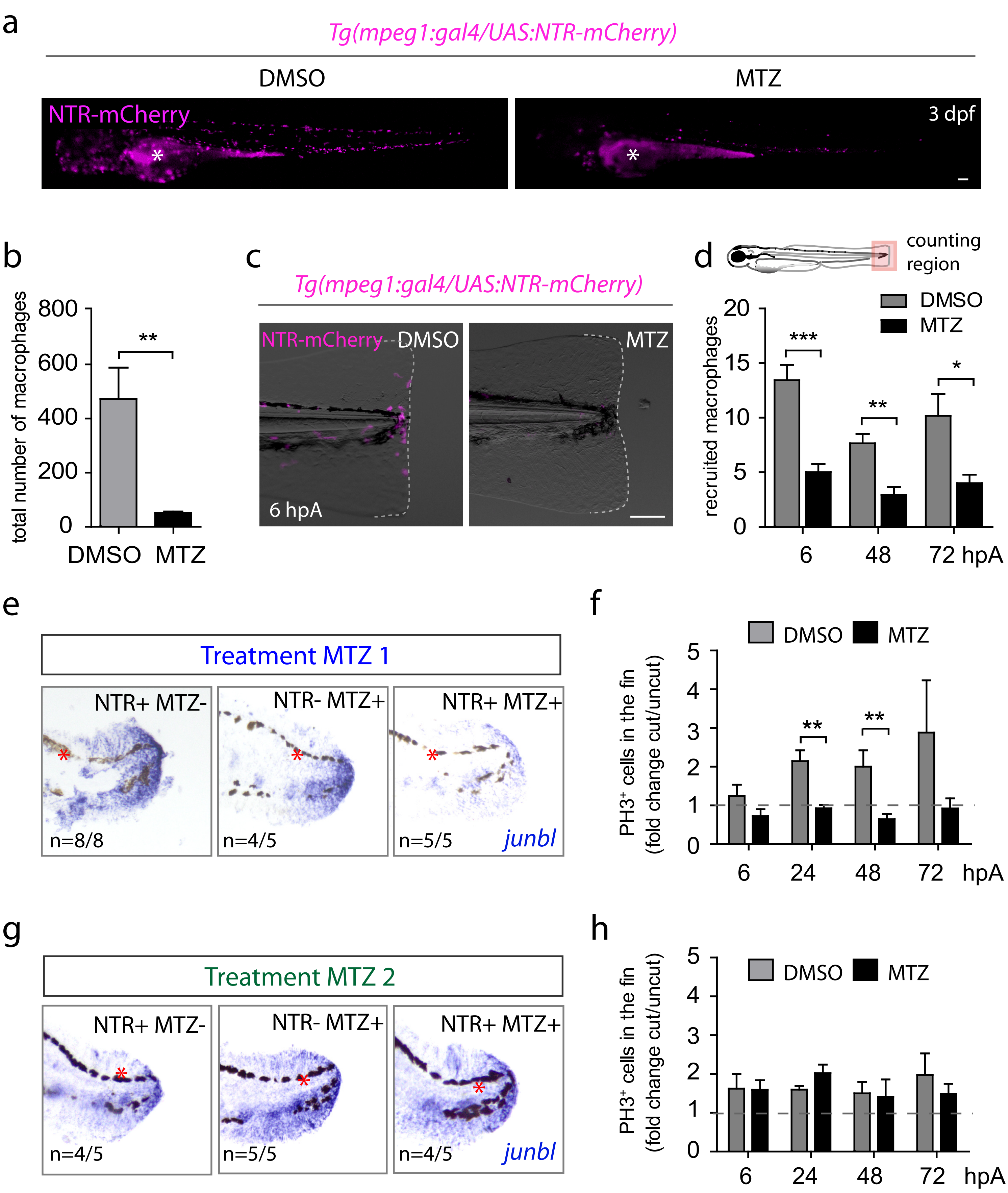

a
Tg(mpeg1:gal4/UAS:NTR-mCherry)
DMSO
MTZ
NTR-mCherry
3 dpf
b
total number of macrophages
800
600
400
200
0
**
DMSO
MTZ
c
Tg(mpeg1:gal4/UAS:NTR-mCherry)
NTR-mCherry
DMSO
MTZ
6 hpA
d
counting region
DMSO
MTZ
recruited macrophages
20
15
10
5
0
***
**
*
6
48
72 hpA
e
Treatment MTZ 1
NTR+ MTZ-
NTR- MTZ+
NTR+ MTZ+
n=8/8
n=4/5
n=5/5
junbl
f
DMSO
MTZ
PH3⁺ cells in the fin
(fold change cut/uncut)
5
4
3
2
1
0
**
**
6
24
48
72
hpA
g
Treatment MTZ 2
NTR+ MTZ-
NTR- MTZ+
NTR+ MTZ+
n=4/5
n=5/5
n=4/5
junbl
h
DMSO
MTZ
PH3⁺ cells in the fin
(fold change cut/uncut)
5
4
3
2
1
0
6
24
48
72
hpA

Figure S4

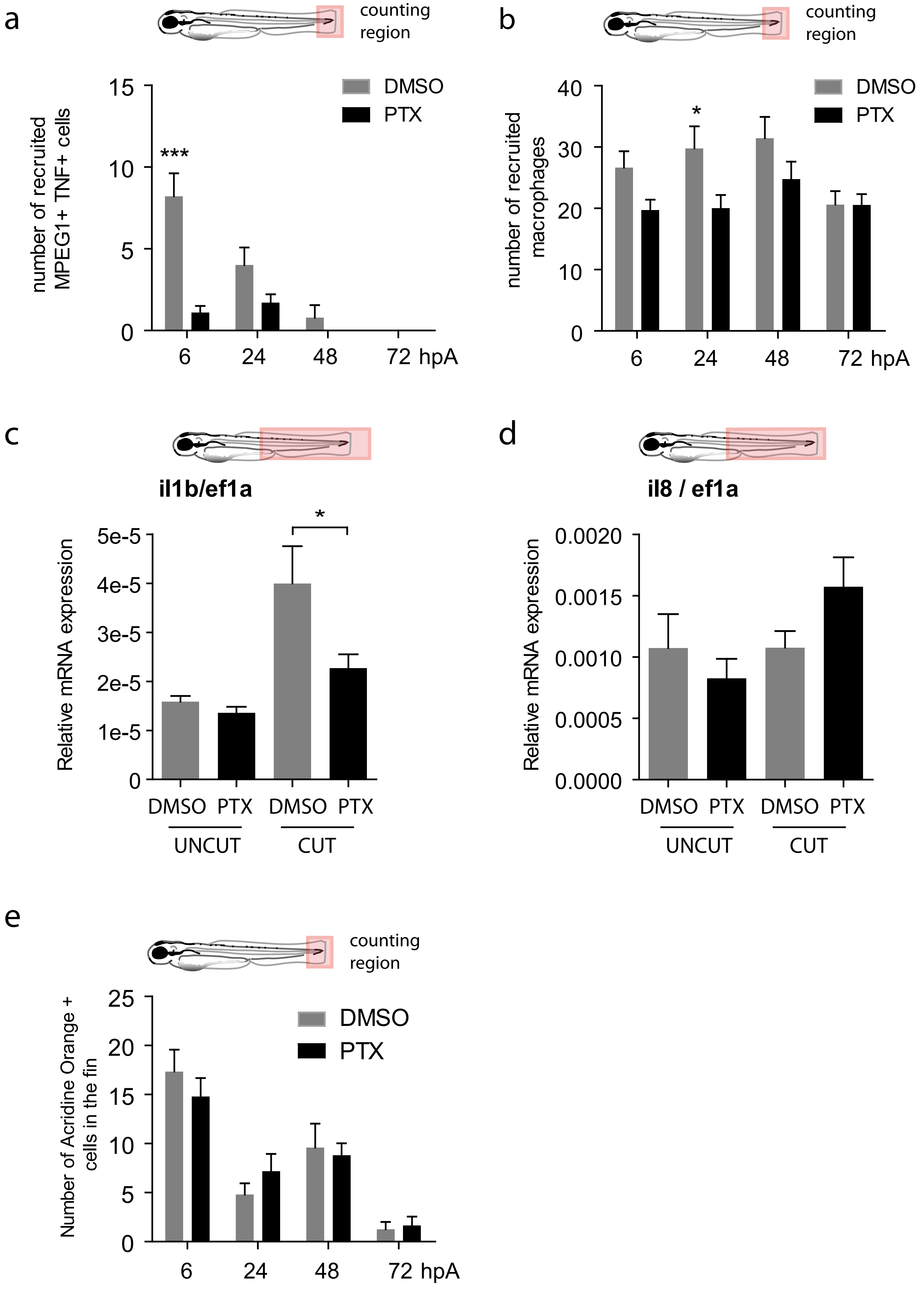
a
counting
region
15
10
5
0
number of recruited
MPEG1+ TNF+ cells
***
DMSO
PTX
6
24
48
72 hpA
b
counting
region
40
30
20
10
0
number of recruited
macrophages
*
DMSO
PTX
6
24
48
72 hpA
c
il1b/ef1a
5e-5
4e-5
3e-5
2e-5
1e-5
0
Relative mRNA expression
*
DMSO PTX DMSO PTX
UNCUT
CUT
d
il8 / ef1a
0.0020
0.0015
0.0010
0.0005
0.0000
Relative mRNA expression
DMSO PTX DMSO PTX
UNCUT
CUT
e
counting
region
25
20
15
10
5
0
Number of Acridine Orange +
cells in the fin
DMSO
PTX
6
24
48
72 hpA

Figure S5

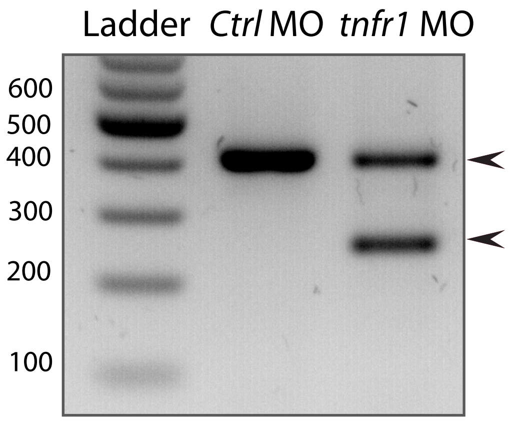

Figure S6

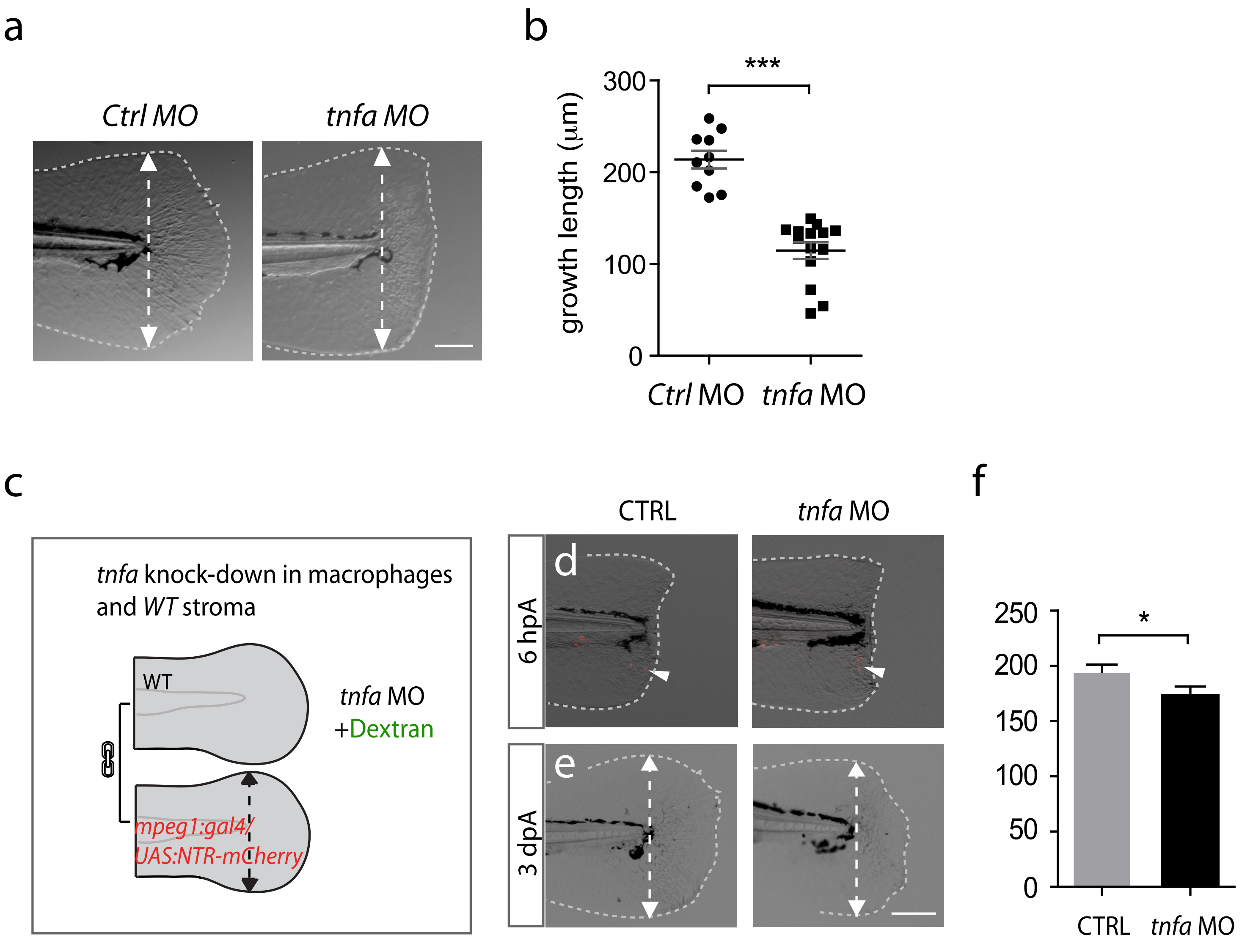
a
Ctrl MO
tnfa MO
b
growth length (µm)
300
200
100
0
***
Ctrl MO
tnfa MO
c
tnfa knock-down in macrophages and WT stroma
WT
tnfa MO
+Dextran
mpeg1:gal4/
UAS:NTR-mCherry
CTRL
tnfa MO
d
6 hpA
e
3 dpA
f
250
200
150
100
50
0
*
CTRL
tnfa MO

Supplementary Figure Legends

Supplementary Figure 1: L-clodronate injections in *Tg(mpeg1:mCherry-F)* larvae efficiently ablate macrophages.

(a) Double transgenic larvae *Tg(mpx:eGFP/mpeg1:mCherry-F)* were injected with either L-PBS or L-clodronate in the caudal vein at 48 hpf. eGFP (Green) and mCherry (Magenta) fluorescence were analyzed by microscopy at 3 dpf. Larva images are representative single fluorescence channels and overlay images (Merge). Asterisks show the auto-fluorescence of the yolk sac and extension. (b) Quantification of total macrophages in *Tg(mpeg1:mCherry-F)* 24 hours after L-PBS or L-clodronate (L-clo) injections using fluorescence microscopy (mean ± s.e.m, N_{larvae}=5, **p<0.01). (c) Graphed data of fluorescence-activated flow cytometry analysis of *mpeg1*$^{+}$ and *mpx*$^{+}$ cells 24 hours after injection of L-clodronate or L-PBS. *Tg(mpeg1:mCherry-F/mpx:eGFP)* larvae were either injected with L-PBS or with L-clodronate at 2 dpf and cells from a pool of 40 larvae were collected 24 hours later. Gates represent mCherry-F^{+} and eGFP^{+} populations (the percentage among total cells is indicated). (d) *Tg(mpeg1:mCherry-F)* larvae were injected with L-PBS or L-clodronate at 48 hpf and amputated at 3 dpf. Fin images are representative overlay of mCherry fluorescence (magenta) with transmitted light acquired by microscopy at 4 hpA. Dotted lines outline the fin. (e) Corresponding counts of recruited macrophages in the caudal fin region in indicated conditions (mean number of cells ± s.e.m, N_{larvae}=10-12 larvae per groups, ***p<0.001). (a, d) Scale bars = 100 μm.

Supplementary Figure 2: Depletion of macrophages using L-Clodronate injections in early stages but not late stages of regeneration impairs blastema formation.

(a and c) Blastema cell proliferation at 6, 48 and 72 hpA after L-clodronate 1 (a) and L-clodronate 2 (c) treatments in indicated conditions. Mitotic cells were detected using an anti-phosphorylated histone H3 (PH3) antibody (N_{larvae} = 4-6 per group, average value of cut/uncut ratio ± s.e.m, differences are not significant). (b) *junbl* mRNA expression (blue), as shown by *in situ* hybridization, in non-amputated fin or 6 hpA, in *Tg(mpeg1:mCherry-F)* larvae that were previously injected with PBS or L-clodronate 24 hours before amputation.

Supplementary Figure 3: Metronidazol treatments on *Tg(mpeg1:GAL4/UAS:NTR-mCherry)* larvae efficiently ablate macrophages and impair blastema formation in early stages of regeneration.

(a) *Tg(mpeg1:GAL4/UAS:NTR-mCherry)* larvae were treated with either Metronidazol (MTZ) or DMSO at 48 hpf. NTR-mCherry fluorescence (magenta) was analyzed by microscopy 24 hours later (3dpf). Asterisks show the auto-fluorescence of the yolk sac and extension. (b) Corresponding counts of total macrophages in indicated conditions (mean ± s.e.m, N_{arvae}=5-6, **p<0.005). (c) *Tg(mpeg1:GAL4/UAS:NTR-mCherry)* larvae were treated with either MTZ or DMSO at 48 hpf and amputated at 3 dpf. Fin images are representative overlay of NTR-mCherry fluorescence (magenta) with transmitted light acquired by microscopy at 6 hpA. Dotted lines outline the fin. (a, c) Scale bars = 100 μm. (d) Corresponding counts of recruited macrophages in the caudal fin region in indicated conditions (mean number of cells ± s.e.m (N_{larvae}=6-12 per group, ***p<0.001, **p<0.005

and *p<0.05). (e and g) *junbl* mRNA expression (blue) at 24 hpA, as shown by *in situ* hybridization in (NTR^+ MTZ^-), (NTR^- MTZ^+) and (NTR^+ MTZ^+) larvae. *Tg(mpeg1:GAL4/UAS:NTR-mCherry)* larvae were treated with MTZ (NTR^+ MTZ^+). DMSO treatments on the same line (NTR^+ MTZ^-) or MTZ treatments on WT siblings (NTR^- MTZ^+) were used as controls. Larvae were treated with DMSO or MTZ either 24 hours before amputation (Treatment MTZ 1, e) or at 6 hpA (Treatment MTZ 2, g), red asterisks show pigments (black). (f and h) Blastema cell proliferation at 6, 24, 48 and 72 hpA after MTZ 1 (f) and MTZ 2 (h) treatments in indicated conditions using an anti-PH3 antibody (N_{larvae} = 5-7 per group, mean values ± s.e.m, **p<0.01).

Supplementary Figure 4: Effect of pentoxifylline treatment on macrophage recruitment and activation and expression of pro-inflammatory cytokines.

(a-b) *Tg(tnfa:eGFP-F/mpeg1:mCherry-F)* larvae were treated with either DMSO or PTX. (a) $GFP\text{-}F^+$ macrophage counts in the wound region in the indicated conditions. (b) Recruited macrophage counts in the wound region in the indicated conditions. (N_{larvae}=8-10, mean ± s.e.m, two independent experiments, *p<0.05 and ***p<0.001). (c-d) qRT-PCR analysis of the steady state of *il1b* (c) and *il8* (d) mRNAs in DMSO or PTX treated larvae (relative to *ef1a*). Caudal fins were either uncut or amputated (cut) at 3 dpf and larvae were immediately treated with DMSO or PTX. RNA was extracted from tails (18 larvae per sample) at 5 hpA. Graph represents mean values ± s.e.m of 3 experiments, *P<0.05. (e) Cell death counts in DMSO or PTX treated larvae (N_{larvae}= 4-6 per group, mean values ± s.e.m, differences are not significant). Cell death was detected using

Acridine Orange staining in DMSO or PTX treated larvae in which late macrophages were depleted and confocal imaging at 6, 24,48 and 72 hpA.

Supplementary Figure 5: tnfr1 morpholino alters the RT-PCR pattern of tnfr1. Efficient blocking of *tnfr1* mRNA splicing upon *tnfr1* MO injection. PCR amplification of *tnfr1* cDNA from the 3 dpf larvae previously injected with either Control morpholino (*Ctrl* MO) or with the morpholino anti-sense oligonucleotide that blocks the splicing of *tnfr1* mRNA (*tnfr1* MO).

Supplementary Figure 6: Effect of *tnfa* morpholino on caudal fin regeneration

(a) Fin images are representative transmitted light images of *Ctrl* morphants and *tnfa* morphants at 3dpA. (b) Corresponding regenerated fin length (mean ±s.e.m. from three independent experiments, ***P<0.001). (c) Schematic representation of the parabiosis experiment using *tnfa* MO or WT (CTRL) and *Tg(mpeg1:GAL4/UAS:NTR-mCherry)*. Parabiotic larvae were treated with MTZ at 48 hpf and the caudal fin of *Tg(mpeg1:GAL4/UAS:NTR-mCherry)* was amputated at 3 dpf. (d) Representative fin images of mCherry fluorescence merged with transmitted channel at 6 hpA show NTR-mCherry^{+} macrophages that are mainly depleted. White arrowheads show residual fluorescence in cells or cell fragments. (e) Fin images are representative transmitted light images at 3 dpA in *tnfa* morphants and CTRL. (f) Corresponding quantification of the regenerated fin length in indicated conditions (N_{larvae}=8-10 mean ± s.e.m., *P<0.05). In (a, d and e) dotted lines outline the fin, dashed arrows indicate the position of the initial transection. Scale bars = 100 μm.
